# The *in crystallo* optical spectroscopy toolbox

**DOI:** 10.1107/S1600576725003541

**Published:** 2025-05-31

**Authors:** Nicolas Caramello, Virgile Adam, Arwen R. Pearson, Antoine Royant

**Affiliations:** a European Synchrotron Radiation Facility 71 Avenue des Martyrs CS 40220 38043Grenoble Cedex 9 France; b Institute for Nanostructure and Solid State Physics Universität Hamburg, Center for Ultrafast Imaging HARBOR, Bldg 610, Luruper Chaussee 149 Hamburg22761 Germany; c Univ. Grenoble Alpes CNRS CEA Institut de Biologie Structurale (IBS) 71 Avenue des Martyrs CS 10090 38044Grenoble Cedex 9 France; Uppsala University, Sweden; The European Extreme Light Infrastructure, Czechia

**Keywords:** *in crystallo* spectroscopy, macromolecular crystallography, time-resolved crystallography, optical spectroscopy, absorption spectroscopy, protein crystals

## Abstract

Over the past ten years, there has been a surge in the demand for *in crystallo* optical spectroscopy (*ic*OS), since optical spectroscopy is one of the few biophysical characterization methods applicable to both protein solutions and crystals. Historically, *ic*OS has been used to compare the state of proteins in crystals and in solution, and to assess their functionality by determining the redox state of metal ions, cofactors or chromophores. The recent rejuvenation of time-resolved crystallography experiments has sparked a renewed interest in optical spectroscopy as a bridge between kinetic studies in solution and in the crystalline state. The method of *ic*OS can be defined as the ensemble of spectroscopic techniques in the UV–visible–infrared range that can be applied to crystals. It has also been instrumental in understanding specific X-ray radiation damage to redox-sensitive parts of proteins. Spectra recorded from crystals are affected by crystal orientation, shape or position due to various optical phenomena. Fortunately, these can be modelled and their effect can be corrected. The *ic*OS laboratory at the European Synchrotron Radiation Facility (ESRF) specializes in recording UV–Vis absorption, fluorescence emission and Raman spectra from protein crystals. Here, we present a suite of utilities that streamline the analysis and correction of UV–Vis absorption *ic*OS data, encased in a graphical interface. This was originally developed for the *ic*OS laboratory at ESRF but is available as a standalone package, with the aim of making *ic*OS more accessible.

## Abstract 2

The rise of time-resolved macromolecular crystallography has been accompanied by renewed interest in companion biophysical characterization methods applicable to molecules both in solution and *in crystallo*. Here, we present a workflow and a graphical interface for analysing spectroscopic data collected on macromolecular crystals.

## 1. Introduction

*In crystallo* optical spectroscopy (*ic*OS) was born of the need for the first protein crystallographers to assess how comparable their crystal structures were to the conformations of biomolecules in solution (Mozzarelli & Rossi, 1996 ▸). This validation was achieved by performing kinetic assays using UV–Vis absorption spectroscopy (AS) on a suspension of crystals instead of a protein solution. AS studies conducted on slurries of crystals within their mother liquor validated that ribonuclease S (Doscher & Richards, 1963 ▸), α-chymotrypsin (Kallos, 1964 ▸) and carb­oxy­peptidase A (Quiocho & Richards, 1966 ▸), as well as many other enzymes both at physiological and sub-zero temperatures (Fink & Ahmed, 1976 ▸; Makinen & Fink, 1977 ▸), were active in the crystalline state. AS spectra of solutions were recorded in UV–Vis absorption spectrophotometers producing millimetre-sized collimated light beams traversing quartz cuvettes. Because of the beam size, such devices are not suited for single macromolecular crystals, and crystal suspensions needed to be used, resulting in significant light scattering and spectra with poor signal-to-noise ratio. Microspectrophotometers were developed to record AS from single crystals by focusing the incident beam down to a few micrometres (Hadfield & Hajdu, 1993 ▸). This allowed the recording of spectra from the same sample that was used for diffraction, either directly at the beamline or in a nearby off-line facility (Pearson *et al.*, 2004 ▸). Since then, *ic*OS has become the technique of choice for functionally characterizing crystallized biomolecules such as metalloproteins (Berglund *et al.*, 2002 ▸; Rose *et al.*, 2024 ▸), fluorescent proteins (Royant *et al.*, 2007 ▸; De Zitter *et al.*, 2020 ▸), photoactive proteins (Edman *et al.*, 1999 ▸; Kovalev *et al.*, 2023 ▸) and enzymes with coloured cofactors (Orru *et al.*, 2011 ▸; Safari *et al.*, 2023 ▸).

Interest in tracking the molecular details of biological processes via X-ray crystallography soon evolved into a wide range of methods designed to characterize reaction intermediate states (RIS), either by chemically or thermally altering a reaction (Fink & Ahmed, 1976 ▸; Edman *et al.*, 1999 ▸) or by recording time-resolved Laue diffraction patterns on the fly during a reaction, using isolated electron bunches at synchrotron sources (Šrajer *et al.*, 1996 ▸). *ic*OS was key to both the experimental design and the validation of the trapped or caught RIS (Bourgeois & Royant, 2005 ▸). However, it rapidly became apparent that the X-ray radiation used for the measurement itself was altering the structure of the biomol­ecules in the crystal (Ravelli & McSweeney, 2000 ▸). This soon became a sizeable issue for kinetic crystallography experiments when it was demonstrated that X-ray-induced features around redox-sensitive moieties could be mistaken for RIS (Matsui *et al.*, 2002 ▸). *In crystallo* UV–Vis absorption spectroscopy (*ic*AS) (McGeehan *et al.*, 2009 ▸) and Raman spectroscopy (Carpentier *et al.*, 2010 ▸) were leveraged to assess both the nature and the extent of the effect of the radicals generated in protein crystals. While originally identified in cryo-cooled crystal structures, specific radiation damage remains a concern for diffraction data obtained at room temperature (Nave & Garman, 2005 ▸; Garman, 2010 ▸; Garman & Weik, 2023 ▸), although its impact on the resulting electron-density map depends on the single-crystal or serial-crystallography nature of the experiment (Gotthard *et al.*, 2019 ▸; de la Mora *et al.*, 2020 ▸).

Time-resolved macromolecular crystallography (TR-MX) experienced a renaissance when it was demonstrated that the femtosecond-long X-ray pulses created by X-ray free electron laser sources could produce diffraction images from single micro- or nano-crystals (Neutze *et al.*, 2000 ▸; Chapman *et al.*, 2011 ▸). The resulting time-resolved serial-crystallography methodology has since been applied to study many different macromolecular systems, predominantly those that could be activated by light (Brändén & Neutze, 2021 ▸). This developing field builds on the knowledge of reaction kinetics in solution obtained from transient and steady-state optical spectroscopy. For example, the RIS identified in the first ultrafast serial femtosecond crystallography study on photoactive yellow protein (Pande *et al.*, 2016 ▸) were named after the intermediates identified via ultrafast spectroscopy studies (Lincoln *et al.*, 2012 ▸). Recording a crystallographic dataset of a reaction intermediate state by serial crystallography requires large amounts of the protein sample; hence, when possible, these experiments are often planned using information from preliminary biophysical studies. Spectroscopy, when available, is becoming the biophysical technique of choice for both the validation and the planning of transient-state structural characterization (Nass Kovacs *et al.*, 2019 ▸).

Importantly, the densely packed environment of a crystal can hinder or even prevent the movements required for a protein to perform its function. Very often, reaction kinetics are altered *in crystallo*. This can be because of the crystal packing itself (Kort *et al.*, 2003 ▸; Konold *et al.*, 2020 ▸; Aumonier *et al.*, 2022 ▸), the hydration level (Efremov *et al.*, 2006 ▸; Konold *et al.*, 2020 ▸) or a crystallization pH different from that of the in-solution studies (Makinen & Fink, 1977 ▸; Mozzarelli & Rossi, 1996 ▸). The presence of viscous precipitants such as polyethyl­ene glycol can also alter kinetics in proteins (Saxena *et al.*, 2005 ▸). Because of this, whenever possible, reaction timescales must also be assessed *in crystallo*. The assumption that the crystal packing, crystallization condition or slow cryo-trapping technique has not driven the reaction off the physiological pathway must be verified (Wilmot *et al.*, 2002 ▸; Caramello & Royant, 2024 ▸). This can be achieved using a complementary biophysical characterization technique that can be applied both *in crystallo* and in solution, such as *ic*OS.

TR-MX experiments relying on photoactivation usually require pulsed laser sources. Because macromolecular crystals are optically dense, high peak fluences are often used to ensure activation of a significant proportion of the molecules in the crystal so that RIS can be detected in the diffraction data. Using these high peak fluences increases the likelihood of two-photon absorption in parts of the crystal. This possibility must be evaluated, as multiphoton absorption can lead to artefactual reaction pathways (Barends *et al.*, 2024 ▸). Indeed, artefacts created by pump laser intensity have been observed for reactions in crystals both by time-resolved *ic*OS (TR-*ic*OS) AS (Engilberge *et al.*, 2024 ▸) and by TR-MX (Barends *et al.*, 2024 ▸; Bertrand* et al.*, 2024 ▸).

The method of *ic*OS can also help estimate the occupancy of a reaction intermediate state over time or with respect to pump light fluence (Engilberge *et al.*, 2024 ▸). This information is crucial to guide the refinement of TR-MX structures and is particularly relevant for structure-factor extrapolation (De Zitter *et al.*, 2022 ▸; Vallejos *et al.*, 2024 ▸).

Recording meaningful *ic*OS data, with minimal optical artefacts, requires knowledge of the setup used and its limitations. Such spectroscopy setups are scarce and often only available only for short beamtimes. In this article, we describe the optical artefacts affecting *in crystallo* UV–Vis absorption and fluorescence, and we present the *ic*OS toolbox, a user-friendly graphical application aimed at correcting such artefacts as well as providing live quality assessment and analysis of *ic*OS data.

## 2. Challenges for *in crystallo* absorption spectroscopy

### 2.1. Background subtraction

**Figure 1:**
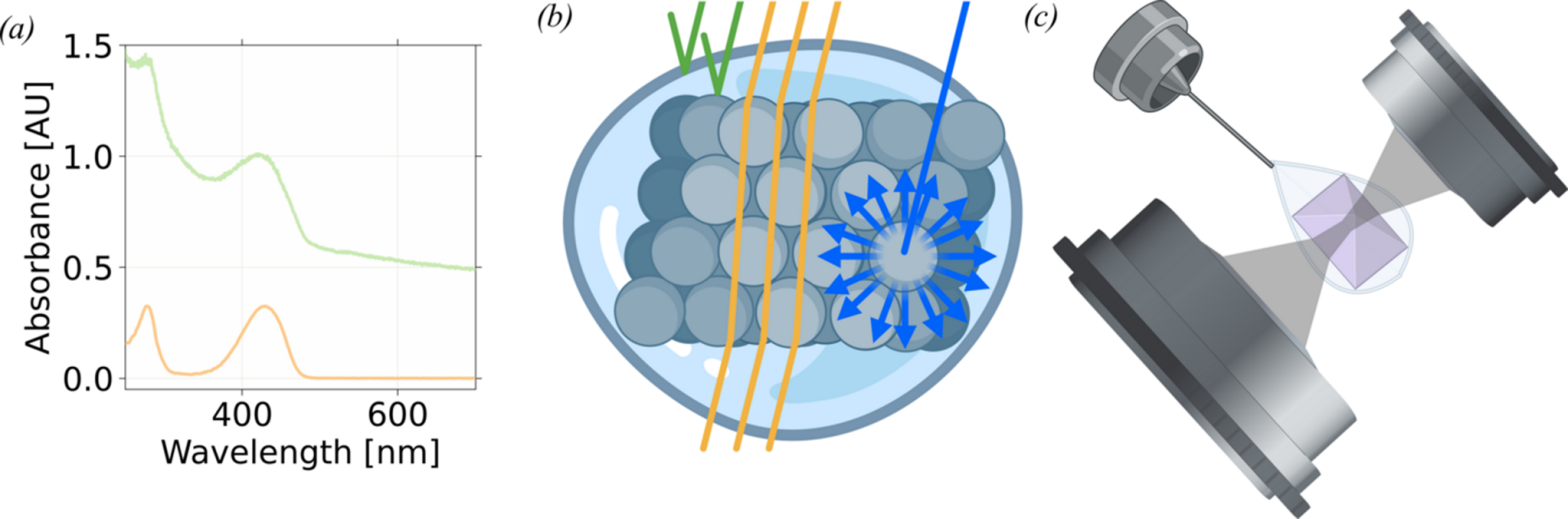
Optical phenomena occurring in a protein crystal. (*a*) Comparison of in-solution (orange curve) and *in crystallo* (green curve, with a non-null non-flat baseline) AS spectra. (*b*) Representation of several optical phenomena occurring in crystals that can contribute to the background: reflectivity (green rays), refraction (orange rays), which does not *per se* cause photon loss, and Rayleigh scattering (blue rays), which causes photon loss. (*c*) Schematic representation of a single-crystal microspectrophotometry setup. The crystal is mounted in a loop and surrounded by its mother liquor. The focal cones of the upstream (top right, focusing the incident beam) and downstream (to the spectrophotometer, bottom left) objectives are shown in grey. The downstream focal cone is larger than the upstream one to mitigate the displacement of the focal-point effect described by von Stetten *et al.* (2015 ▸).

In AS, the baseline of a spectrum is defined by one or several wavelength ranges where the sample does not have significant absorption. Using a reference cuvette in which the molecule of interest is missing allows for the subtraction of the contribution of the sample holder and solvent components. Thus solution spectra of non-turbid solutions (*e.g.* where there is no aggregation when the protein of interest is added or during the studied reaction) recorded in cuvettes typically exhibit flat baselines [Fig. 1 ▸(*a*), orange curve]. Such a subtraction is not possible for *ic*AS since the sample holder cannot be mimicked, as it consists of a complex ensemble of a loop, a mother-liquor droplet and a crystal scaffold. The latter particularly contributes to loss of transmitted photons via diverse optical phenomena.

Due to the high density of scattering material in biomacromolecular crystals, as well as the grating effect of their surface, the quantity of photons diverted from the downstream (after the sample) objective focal cone is much higher in crystals than in solution (Dworkowski *et al.*, 2015 ▸; von Stetten *et al.*, 2015 ▸). This loss, and associated apparent increase in absorption throughout the spectrum, can be broken down into distinct phenomena: reflectivity and refraction of light at interfaces (Cole *et al.*, 1995 ▸), and diffuse Rayleigh scattering [Fig. 1 ▸(*b*)]. Reflectivity and refraction depend on the refractive indices of the various media and the angle of the incident light ray. Refraction of light at the interfaces can be neglected with the reasonable hypothesis that the refractive index of the crystal is constant throughout and that its faces are parallel [Fig. 1 ▸(*b*)], but reflectivity cannot. The refractive index of a medium is a function of the wavelength of the incoming light. Therefore, achromatic reflectivity will contribute to the background of the *ic*AS spectrum. In order to minimize reflectivity, the crystal should be aligned to present a flat face to both incoming and downstream objective. This orientation usually yields the ‘flattest’ baseline. Rayleigh scattering occurs when the incoming light interacts with particles that are much smaller than the wavelength of the light (Lahiri, 2016 ▸). The incoming photons are elastically scattered in a random direction [Fig. 1 ▸(*b*)]. Rayleigh scattering scales inversely proportional to wavelength (λ) with a 1/λ^4^ dependence, resulting in an increasing number of photons scattered away from the measuring objective as the wavelength decreases. Consequently, the baseline of an *ic*AS spectrum is not flat but instead progressively increases as the wavelength decreases [Fig. 1 ▸(*a*), green curve]. In order to make the *ic*AS spectrum comparable to the corresponding solution spectrum, this background must be modelled and subtracted from the observed signal.

An additional contribution to the absolute absorption measured is the crystal size, or variation in the thickness of material probed by the incoming light. The height of the absorbance peaks and signal-to-noise ratio increase for bigger crystals, up to the point where the peak of interest can become saturated because all of the incoming light at that particular wavelength is absorbed.

### 2.2. Focal-spot displacement

The light beam used for in-solution AS is collimated, whereas it must be focused on the crystal for *ic*AS. Refraction of light at the interfaces of both the crystal and its mother liquor displaces the focal point of the objective, contributing to photon loss (von Stetten *et al.*, 2015 ▸). This effect artificially increases the measured optical density in *ic*AS across the whole spectrum with a ‘flat baseline’ [Fig. 1 ▸(*a*)]. This effect can be mitigated by using a larger-diameter optical fibre for the downstream objective than for the upstream objective, thereby increasing the downstream focal cone and catching most stray refracted photons [Fig. 1 ▸(*c*)].

### 2.3. Spectral anisotropy

The anisotropic properties of protein crystals also cause spectral artefacts. Photons are maximally absorbed when their electric vector (polarization) is parallel to the dipole transition moment of a chromophore (Eaton & Hofrichter, 1981 ▸). In a solution with randomly oriented chromophores, this does not matter, as photons polarized in any direction are absorbed with an equal probability. This is no longer true in protein crystals, because the chromophores are oriented along a restricted number of directions as a result of the crystallographic and non-crystallographic symmetry axes (Eaton & Hofrichter, 1981 ▸). As a consequence, protein crystals exhibit different extinction coefficients depending on crystal orientation with respect to the incident light beam (Eaton & Hochstrasser, 1968 ▸), thus breaking the Beer–Lambert law. An extreme example of optical anisotropy can be observed for the orange carotenoid protein (OCP), where the two carotenoid chromophores are positioned almost parallel to each other in the asymmetric unit (Kerfeld *et al.*, 2003 ▸). If observed through a polarized filter, OCP crystals appear totally colourless in some orientations and orange in others; in other words, the crystals are birefringent (Kerfeld *et al.*, 1997 ▸). While OCP is an extreme example, all protein crystals exhibit anisotropic optical properties to varying degrees. Because the protein crystal also acts as a light polarizer, the shape of an *ic*AS spectrum will depend strongly on the crystal orientation. In order to record quantitative absorbance in crystals, the incoming and downstream light must be polarized along one of the symmetry axes of the crystals. In this case, the probability of absorption is equal for all photons and the Beer–Lambert law is again applicable (Mozzarelli & Rossi, 1996 ▸).

### 2.4. Artefacts associated with fluorescence

**Figure 2:**
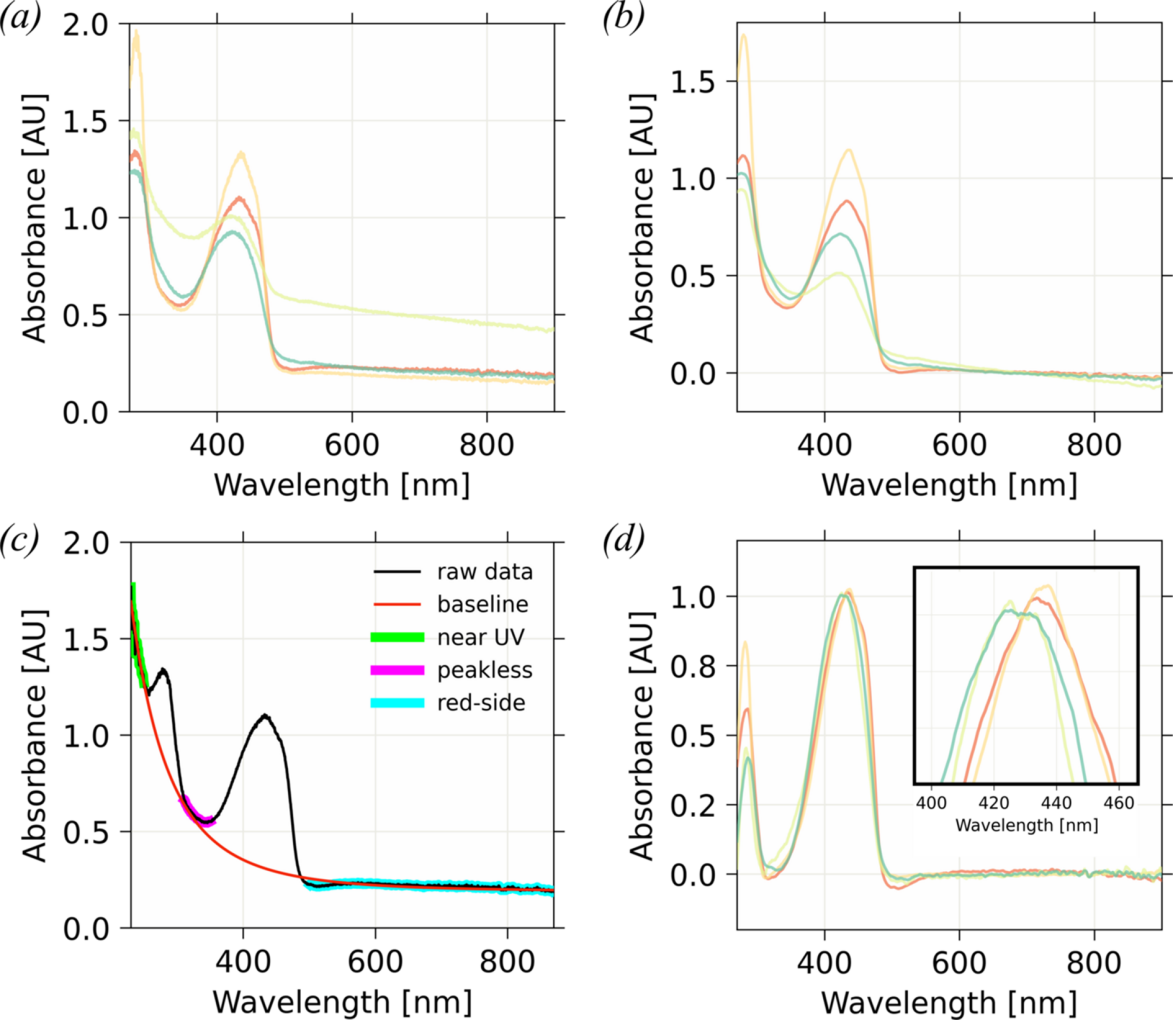
The *ic*AS spectra of different crystals of the Cerulean fluorescent protein, at several points after they were soaked from pH 8.0 to pH 4.0 (red to blue–green). (*a*) Without correction and (*b*) after constant-baseline correction and smoothing. (*c*) Example of baseline and scattering baseline correction of the red spectrum from (*a*), with the three segments used to fit the baseline: red-side baseline segment (cyan), peakless segment (magenta) and near-UV segment (lime). (*d*) Baseline and scattering corrected spectra, scaled according to their absorption at 430 nm for comparison purposes. The correction separates the red and yellow spectra from the dark-green and light-green spectra. The inset shows the detail of the peak summit, 390 to 450 nm.

Other phenomena can unfortunately not be reliably modelled and corrected. Owing to the density of fluoro­phores in protein crystals (including aromatic amino acids), a significant part of the absorbed light will generate fluorescent photons, some of which are collected by the measuring objective, creating dips in measured absorbance (visible in the red spectrum represented in all parts of Fig. 2 ▸) (von Stetten *et al.*, 2015 ▸). Conversely, when measuring fluorescence emission spectra, the focusing volumes of the cones for both the excitation light and the measured signal need to be carefully chosen to prevent the ‘self-absorption’ phenomenon leading to apparent red shifts of emission maxima (Barros *et al.*, 2009 ▸; von Stetten *et al.*, 2015 ▸).

## 3. Correction of *in crystallo* UV–Vis absorption spectra in the *ic*OS toolbox

The example spectra analysed here have been recorded on various instruments on beamlines at the European Synchrotron Radiation Facility (ESRF) or on off-line setups nearby: the *ic*OS laboratory at the ESRF (von Stetten *et al.*, 2015 ▸) or the CAL(AI)^2^DOSCOPE at the Institut de Biologie Structurale (IBS) (Byrdin & Bourgeois, 2016 ▸). The CAL(AI)^2^DOSCOPE has been specifically designed, though not exclusively, for the study of fluorescent proteins (De Zitter *et al.*, 2019 ▸, 2020 ▸).

**Figure 3:**
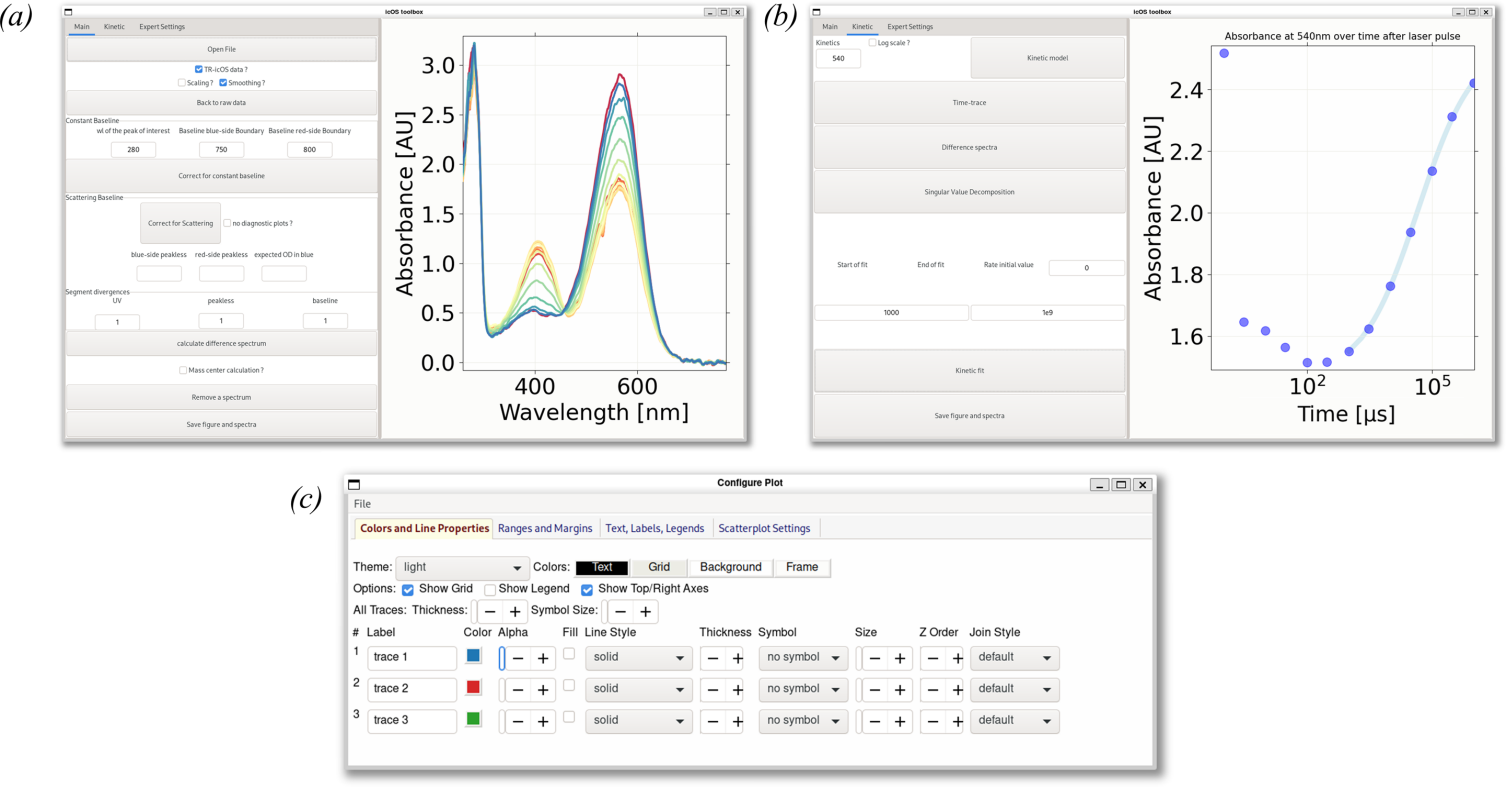
The *ic*OS app GUI – (*a*) main panel, (*b*) kinetic analysis panel and (*c*) figure-customization panel.

In these setups, a ‘dark’ reference is first recorded (*I*_background_) with the white lamp turned off, corresponding to the background counts of the CCD camera. Then, with the lamp turned on, the reference light signal (*I*_reference_) is recorded, without any sample present in the optical path. Finally, after the sample is mounted and aligned, the experimental signal (*I*_sample_) is recorded, which consists of the photons transmitted through the sample. Accordingly, absorbance is calculated as follows:
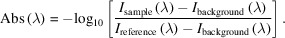


All the phenomena discussed above complicate the direct comparison of *ic*AS data with in-solution AS data. Before an *ic*AS spectrum is measured, the orientation of the crystal must be carefully chosen to minimize the effect of focal spot displacement, anisotropy and potentially fluorescence. Additionally, contributions from Rayleigh scattering, focal-spot displacement and reflection must be modelled to allow *ic*AS data recorded on different crystals to be compared. Here, we present a workflow for these correction procedures. The Python application described here is wrapped in a graphical interface [Fig. 3 ▸(*a*)] able to apply these corrections to *ic*AS and *in crystallo* fluorescence spectra, as well as analyse and compare spectra and prepare publication-ready figures.

Several types of background and data correction are available, each of them addressing a specific optical artefact. In addition, the *ic*OS toolbox provides a tool for live kinetic analysis of the data [Fig. 3 ▸(*b*)]. Finally, figures are generated using the *wxmplot* package (Newville, 2024 ▸), and can be fully customized to reach publication quality [Fig. 3 ▸(*c*)]. The corrected data can be saved to ASCII format. This software is already deployed on the main setup of the *ic*OS laboratory at the ESRF [HR2000+ or QE65Pro spectrophotometer (Ocean Optics), spectra recorded using *SpectraSuite* (Ocean Optics)], on the TR-*ic*OS instrument (Engilberge *et al.*, 2024 ▸) [AvaSpec-ULS2048CL-EVO-RS-UA spectrophotometer (Avantes), spectra recorded using *AvaSoft* v 8.11 (Avantes)], and on the BM07-FIP2 on-line microspectrophotometry setup [QE65Pro spectrophoto­meter (Ocean Optics), spectra recorded using *Ocean­View* (Ocean Optics)] and the CAL(AI)^2^DOSCOPE setup at IBS. It is available for off-line use at https://github.com/ncara/icOS and has been adapted to process solution data from JASCO spectrophotometers as well as on-line *ic*AS data recorded on beamline I24 at Diamond Light Source (Rose *et al.*, 2024 ▸). It can also be used to treat data recorded on solutions and on small-molecule crystals.

### 3.1. Levelling and scaling: correcting optical density and baseline level

Various phenomena previously described contribute to uniformly raising the baseline of *ic*AS spectra [Figs. 1 ▸(*a*) and 2 ▸(*a*)]. This is easily corrected, provided the spectrum features a region devoid of absorption. The average of absorption in this band is subtracted from each spectrum to bring them onto a common baseline [Fig. 2 ▸(*b*)]. In the app, this function is called ‘constant-baseline correction’.

Unlike solutions in spectroscopy cuvettes, the shape of protein crystals is irregular: they present an optical path of varying depth. The concentration of absorbing species in protein crystals cannot be adjusted and their optical density is anisotropic. Therefore, the amount of absorbing material traversed by the incoming light depends not only on the size and shape of the crystal but also on the orientation of the crystal with respect to the light path. Spectra from different crystals or orientations should thus be scaled with respect to a conserved absorption peak. The choice of peak can be inferred from prior knowledge in solution data.

### 3.2. Scattering and reflection correction

Protein crystals are dense optical media, often surrounded by a layer of solution. The amount of light lost to reflectivity at each interface depends on the refraction indices of the crystal and its mother liquor, which are a function of the wavelength. The contribution of reflectivity to the baseline can be estimated by the Fresnel equations. Assuming the spectra have been recorded in the optimal direction, and the angle of incidence of the incoming light is mostly normal, the share of light lost to reflectivity (*R*) as a function of the refractive indices (*n_i_*) of both media at the interface corresponds to
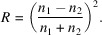
The refractive index of each medium can be expressed as a function of the wavelength (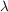) with the Cauchy equations, where *a* and *b* are material-specific coefficients that can be derived by fitting to the measured refractive index at different wavelengths but are used here as parameters fitted against the data to estimate the contribution of reflectivity:
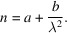
Therefore, the contribution of reflectivity to the baseline distortion of an *ic*AS spectrum can be estimated as
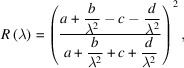
where *a, b, c* and *d* are parameters that will be fitted against the data.

The number of visible-range photons Rayleigh scattered by a protein crystal scales inversely proportional to λ^4^ (Calvert, 1990 ▸):
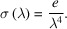
The combined contribution of reflectivity and Rayleigh scattering to the baseline can thus be estimated as a function of the wavelength using
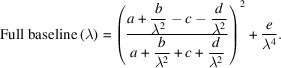


**Figure 4:**
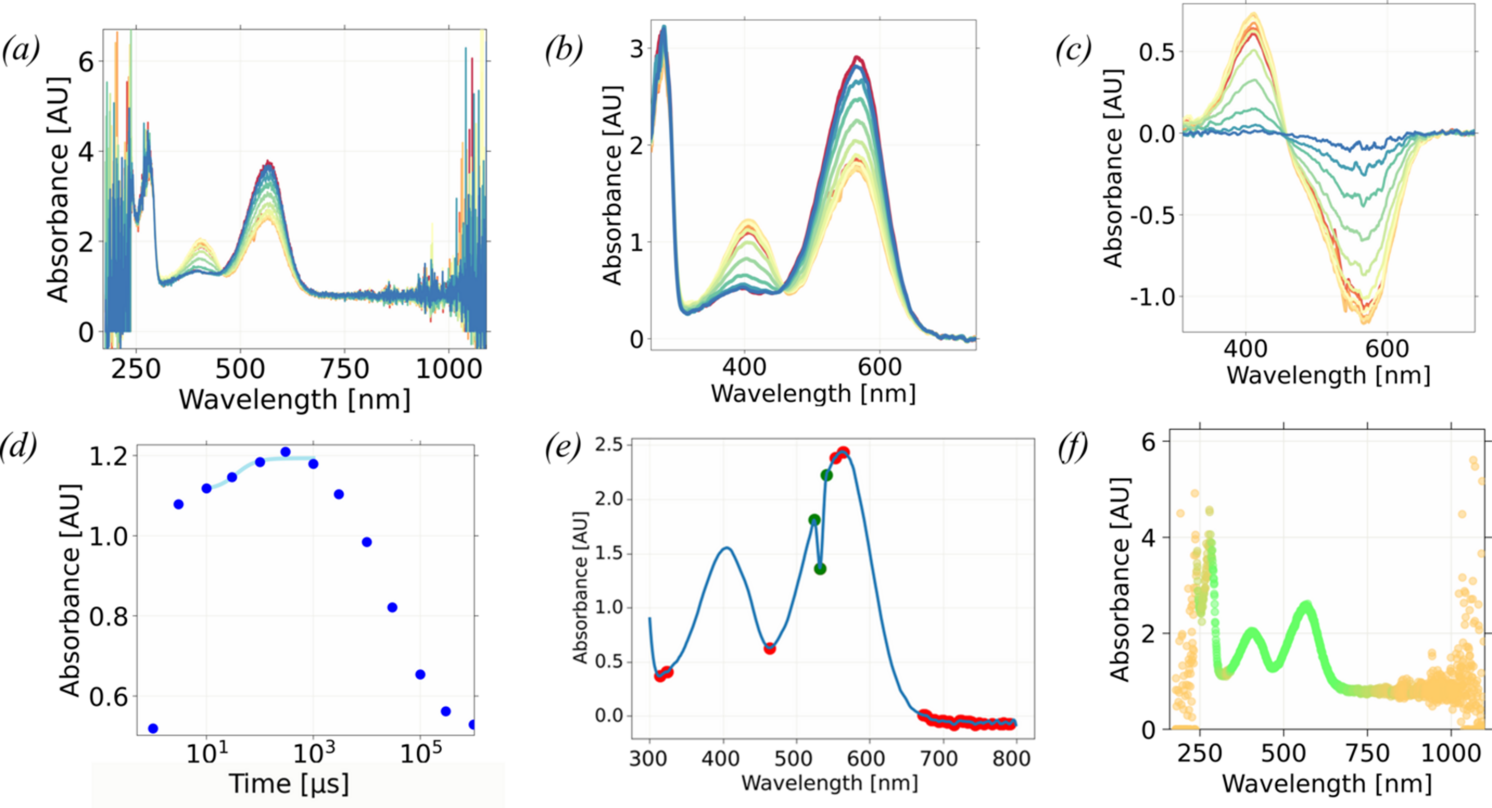
Time-resolved *ic*OS data in the GUI. (*a*) Series of time-resolved AS spectra, collected on BR crystals, after exposure to a 560 nm nanosecond laser pulse, raw. (*b*) Constant-baseline corrected and smoothed spectra, with laser trace removed. (*c*) Series of light − dark difference spectra. For panels (*a*)–(*c*), the colour ramps from red to blue as the reaction progresses, and spectra are recorded at 3 µs, 10 µs, 30 µs, 100 µs, 300 µs, 1 ms, 3 ms, 10 ms, 30 ms, 100 ms, 300 ms and 1 s. (*d*) Absorption at 400 nm (M state of BR), as taken from the spectra presented in (*b*), with the Hill equation fitted to data points from 10 to 1000 µs (pale-blue line). (*e*) Absorption spectrum recorded 3 µs after the actinic laser pulse, in which all dips in absorbance are identified by red dots. The largest dip in absorbance is identified by green dots and corresponds to the tail of the nanosecond laser pulse. (*f*) Confidence plot of the 300 µs TR-*ic*OS spectrum; each data point is plotted from orange (low confidence) to green (good confidence).

In this model, parameters *a*, *b*, *c*, *d* and *e* are fitted against the data points from three non-absorbing (supposed baseline) segments of the spectra via the least-square minimization method [lime, magenta and cyan segments in Fig. 2 ▸(*c*)]. Ideally, two of these segments are on either side of the recorded range [Fig. 2 ▸(*c*)], one where Rayleigh scattering is strongest [lime, Fig. 2 ▸(*c*)] and the other where it is weakest [cyan, Fig. 2 ▸(*c*)]. Because the UV segment is sometimes unreliable [loss of signal through the optics visible in Fig. 4(*a*)], a third segment, between the UV range and the absorption peak of interest [magenta, Fig. 2 ▸(*c*)], is also used to fit the baseline model [plotted in red in Fig. 2 ▸(*c*)]. Additionally, a divergence factor [always positive, 1 by default, as visible in Fig. 3 ▸(*a*)] can be applied to decrease the weight of each segment in the fit of the scattering baseline. This divergence factor should be inversely proportional to the length of its segment and increased if the segment is less reliable. The segments on the left and right side of the region of interest are chosen by the user [boundaries can be filled in the fields visible in Fig. 3 ▸(*a*)]. If the absorbance does not go back to the baseline between the absorption peak of interest and the UV range, a percentage of the maximum absorbance peak can be supplied to create a constant offset between the fit and the absorbance [using the field labeled ‘baseline’ in the ‘Segment divergences’ subsection, Fig. 3 ▸(*a*)]. A diagnostic plot is generated for each spectrum. In this diagnostic plot, segments used in the fit are coloured (lime, magenta and cyan) and the fit baseline is overlaid (red) for assessment of the background-correction quality [Fig. 2 ▸(*c*)]. The range and divergence factor of each segment should be adjusted so that the fit baseline best matches the segments. Finally, the modelled contribution of both phenomena as well as the flat baseline can be subtracted from the raw spectrum, effectively bringing the baseline to 0 [Fig. 2 ▸(*d*)].

The data shown in Fig. 2 ▸ correspond to absorption spectra of crystals of the Cerulean fluorescent protein grown at neutral pH and cryo-cooled at various delays after they were soaked in acidic pH buffer [the behaviour of Cerulean at neutral and low pH is characterized by Gotthard *et al.* (2017 ▸)]. For these spectra, the background correction reveals a blue shift and a change of shape in the main absorbance peak, and allows grouping of spectra into two distinct families: red and yellow spectra with a two-shouldered shape peak at 430 nm, and green and dark-green spectra with a Gaussian shaped peak centred at 425 nm. There is also a change of shape from the initial one-peaked two-shouldered shape [red and yellow spectra, Fig. 2 ▸(*d*)] to a blue-shifted peak without shoulders as time goes on [green spectra, Fig. 2 ▸(*d*)]. The two families of spectra were not so easily discernible in the raw data.

In some cases, such as X-ray-induced baseline alterations (Bolton *et al.*, 2024 ▸), the standard baseline model does not perform well. The user can then choose between pure Rayleigh scattering (no reflectivity) and a custom λ^−*n*^, which, in our experience, has empirically performed well for X-ray-induced optical artefacts.

### 3.3. Smoothing

Due to variations in crystal shape and optical density, the signal-to-noise ratio of *ic*AS data can sometimes be low. Identification of correct peak positions and centre of mass can benefit from a noise-removal step. Both a Savitzky–Golay filter (in this case, by fitting a third-degree polynomial over 21-point windows) and a rolling average are available as options in the ‘expert features’ tab for the smoothing of *ic*OS data.

## 4. Kinetic *ic*OS data analysis

The *ic*OS laboratory allows recording of kinetic series on millisecond to minute timescales for fluorescence decay analysis, monitoring slow protein dynamic events or deriving a photoreduction dose for an X-ray-sensitive species on-line (Bolton *et al.*, 2024 ▸) and off-line (Aumonier *et al.*, 2022 ▸). A recent instrumentation update now allows off-line recording of time-resolved *ic*AS down to the microsecond range (Engilberge *et al.*, 2024 ▸). Depending on the recording procedure, different data-processing pipelines should be used.

### 4.1. Spectral series: the case of time-resolved or dose-resolved data

In an ideal case, a kinetic series is recorded on the same crystal at the same angle and position. Even then, slight wavelength-independent baseline variations can still be observed due to fluctuations of the cryo-stream or humidifier (Sanchez-Weatherby *et al.*, 2009 ▸), or variation in intensity of the pulsed white lamp in the case of the TR-*ic*OS instrument at ESRF. In this ideal case, only the constant-baseline correction described in Section 2.1 is needed, and potentially a smoothing of the data [Figs. 4 ▸(*a*) and 4 ▸(*b*)]. The example shown in Fig. 4 ▸ depicts the correction, smoothing [Fig. 4 ▸(*b*)] and calculation of a series of difference absorption spectra [Fig. 4 ▸(*c*)] of a bacteriorhodopsin (BR) crystal. These corrections allow precise assessment of the timescale of the rise of the characteristic M state 400 nm peak (Efremov *et al.*, 2006 ▸) and the drop in absorbance of the main 600 nm peak as the sample returns to the ground state.

#### 4.1.1. Time trace and constant fitting

Spectral regions corresponding to absorption features of key species can be identified. Absorbance in these regions can be plotted over time in the *ic*OS toolbox graphical user interface (GUI) to create a time trace [Fig. 4 ▸(*d*)]. Provided these prerequisites are met, a kinetic model can be fitted to the data points. The *ic*OS toolbox currently allows the fitting of a mono-exponential decay or rise, as well as the Hill equation. The produced reaction-rate constant provides an estimation of an intermediate-state lifetime and can be used to plan a TR-MX experiment. Finally, rate-constant fits are particularly suited to the detection of artefactual reaction pathways caused by non-linear multi-photon absorption events (Do *et al.*, 2023 ▸; Engilberge *et al.*, 2024 ▸; Barends *et al.*, 2024 ▸; Bertrand *et al.*, 2024 ▸).

#### 4.1.2. Laser dent removal

Because of the duration of integration of the spectrophotometer, the tail of the nanosecond laser pulse used to initiate a reaction in a crystal can also contribute to the absorption spectrum, in the form of a negative dip or dent in the absorption spectrum [Fig. 4 ▸(*e*)]. Local minima of the second derivative of the absorbance spectrum are used to identify both the position and boundaries of the absorption dips. The largest absorption dip (in amplitude) marks the contribution of the nanosecond laser to the spectrum, while all other dips are marked by red dots. The data points corresponding to the contribution of the laser are removed. For spectra of subsequent time points, only the main dent in the area of the previously detected laser dent is marked, and the corresponding data points are also removed.

#### 4.1.3. Confidence score

The polychromatic light sources used for *ic*OS often contain regions of lower photon flux, causing low photon counts in these regions of *I*_reference_. Because of low transmission through the sample, *I*_sample_ also frequently features low-photon-count regions. The signal-to-noise ratio of the calculated absorbance spectrum decreases significantly in regions of low photon counts of either *I*_sample_ or *I*_reference_.

Any photons above the maximum of the dynamic range of the spectrophotometer detector are not measured, distorting the shape of both *I*_reference_ and *I*_sample_, and the resulting calculated absorbance. This is referred to as ‘saturation’ and is often apparent in series of contiguous data points of either *I*_sample_ or *I*_reference_ plateauing at the maximum of the detector dynamic range.

We have implemented a confidence score to highlight, to the user, regions where the absorbance was calculated from critically low or saturated *I*_sample_ and *I*_reference_ photon counts. These regions are shown in orange in the confidence plot [Fig. 4 ▸(*f*)] available from the ‘expert features’ tab of the app, provided the *I*_reference_, *I*_sample_ and *I*_background_ signals have been saved by the spectrophotometer instead of only the absorbance spectrum.

Poor signal-to-noise ratio regions of the absorbance spectrum often exhibit large variations between contiguous data points. It is likely that a criterion of ‘maximal meaningful variation of absorbance’ could be applied to detect these regions. While we have not achieved this yet, we hope to implement this feature in the future development of this software.

#### 4.1.4. Singular value decomposition

When a series of spectra is available, it can be analysed with singular value decomposition (SVD) to provide additional insights into time- or dose-dependent behaviour. SVD is an algebra-based analysis technique that has been widely used in the field of time-resolved spectroscopy (Henry, 1997 ▸; Henry & Hofrichter, 1992 ▸). Briefly, the aim of SVD is to determine a minimal set of basis spectra that can be linearly combined to make up any of the observed spectra across the series. It consists of the decomposition of a matrix, *A*, containing the observed spectra in rows, in chronological order. This input matrix traditionally contains light − dark difference spectra to remove unchanging features. *A* is eigen-decomposed into *U*, *S* and *V*, containing, respectively, the basis spectra (left singular vector, lSV), weighting factors of each feature from *U* (singular values, SVs) and scalars weighting each basis spectrum from *U* to recreate the observed spectra from *A* (right singular vector, rSV). This corresponds, in the case of a time-resolved series of spectra, to a decomposition into bases of time-invariant spectra and time traces of these basis spectra over the studied time range.

**Figure 5:**
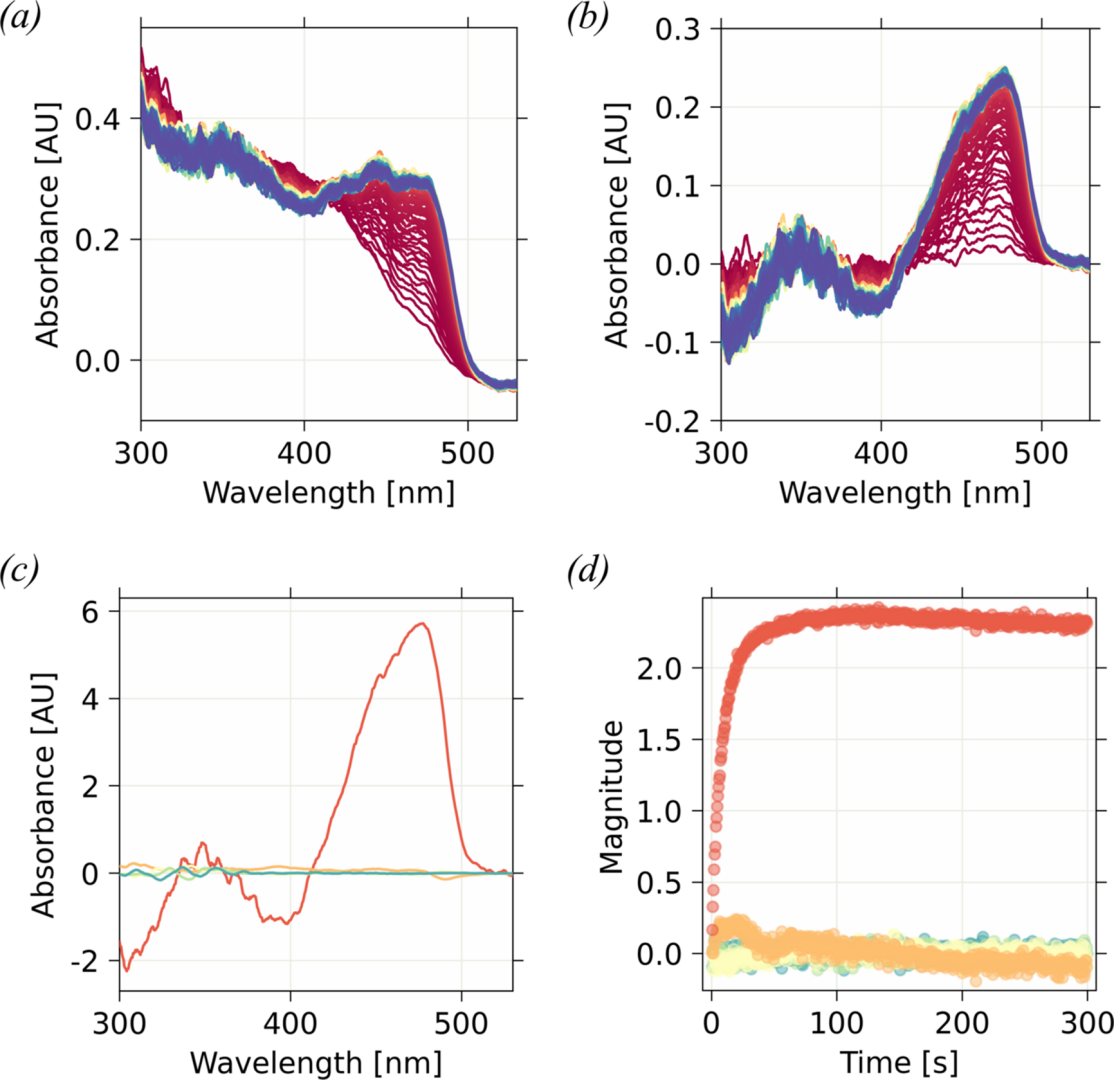
SVD of time-resolved *ic*AS spectra. (*a*) Red to blue, spectra collected on LOV2 crystals, during the relaxation from the photoproduct to the ground state, smoothed and constant-baseline corrected as described by Aumonier *et al.* (2022 ▸). (*b*) Light − dark difference absorption spectra. (*c*) Five first time-invariant components (lSV) resulting from the decomposition of the series of difference spectra: lSV_0_ (red) dominates the series and contains the negative difference absorption band at 390 nm corresponding to photoproduct relaxation, as well as the large positive band spanning from 420 to 500 nm corresponding to ground-state recovery; lSV_1_ (orange) is mostly flat but contains some sparse features from 350 to 510 nm corresponding to systematic background-noise elements; and lSV_2_ (pale yellow), lSV_3_ (pale green) and lSV_4_ (green–blue) only contain non-systematic background noise. (*d*) Time-varying magnitudes (rSV) resulting from the decomposition: rSV_0_ (red) follows the exponential decay kinetics identified by Aumonier *et al.* (2022 ▸); rSV_1_ (orange) follows a linear decay trend, identified as a crystal-displacement component within the loop over the duration of data collection; and rSV_2_ (pale yellow), rSV_3_ (pale green) and rSV_4_ (green–blue) are flat and correspond to background-noise components.

In order to illustrate the potential of SVD analysis, we decomposed a series of time-resolved *ic*AS spectra [Fig. 5 ▸(*a*), red to blue] collected on crystals of the LOV2 domain of phototropin II from *Arabidopsis thaliana* (Aumonier *et al.*, 2022 ▸). This study monitors the structural and spectroscopic relaxation of the LOV2 photoadduct, in which the flavin cofactor forms a covalent bond with a nearby cysteine, into the ground state.

The decomposition of light − dark difference spectra [represented in Fig. 5 ▸(*b*)] produces one main time-invariant component [lSV_0_, red in Fig. 5 ▸(*c*)] with a negative difference peak centred on 390 nm (corresponding to the absorption peak characteristic of the photoadduct) and a large positive peak spanning from 420 to 500 nm (corresponding to the absorption band of the ground state). The time trace of this main time-invariant element [rSV_0_, red in Fig. 5 ▸(*d*)] follows a mono-exponential decay corresponding to the first-order reaction of conversion between two single species. The analysis also reveals a minor time-invariant component [lSV_1_, orange in Fig. 5 ▸(*c*)] that contains systematic background features and whose time trace [rSV_1_, orange in Fig. 5 ▸(*d*)] drifts linearly, on average, over the course of the data collection.

The fact that the main component contains signals from both the photoproduct depletion and the ground-state recovery demonstrates that the two phenomena are simultaneous *in crystallo*, *i.e.* that the photoproduct converts into the ground state without going through another intermediate state, which is consistent with studies in solution (Alexandre *et al.*, 2007 ▸). Moreover, the analysis is sensitive enough to show a quasi-linear component that can be attributed to progressive crystal displacement within the sample support, a typical issue at room temperature, paving the way for optimal spectrum correction.

### 4.2. Serial time-resolved *in crystallo* UV–Vis absorption spectroscopy

For reactions that are too slow or irreversible *in crystallo*, only one measurement per crystal is possible. For measurements performed on the same crystal, only the constant-baseline correction is needed, to account for variation in surrounding-medium refractive index or flash-lamp pulse intensity. After this correction, light − dark difference spectra can be calculated for each time point. In a series of spectra (recorded on different crystals), the height of the difference peaks is influenced by the thickness of each crystal, meaning a scaling scheme has to be applied before comparison. This enables pairwise analysis if the correction of crystal-shape-derived baseline artefacts proves impossible. Here, once bands of interest are identified in the difference spectra, the area under the bands of interest can be integrated and plotted against time, creating a serial-*ic*OS time trace. Because of all the phenomena described in Section 2, it is challenging to translate an integrated absorbance value into the occupancy of a reactive intermediate in this case. However, a kinetic model can be fitted to this set of points, providing a precise estimation of a reaction intermediate rise and decay time *in crystallo*.

## 5. Dos and don’ts

The wavelength, or wavelength range, chosen for the extraction must be devoid of any saturation (shown by noisy truncated peak summits), but must also exhibit reasonably high confidence [see Fig. 4 ▸(*f*)].

Ideally, only the tracked species should absorb in the chosen region, so that a change of absorption can be solely attributed to a change in occupancy of the tracked species. Choosing a good wavelength for the investigation of a reaction intermediate state can mean choosing to minimize noise and contamination over maximizing the intensity of the signal.

## 6. Conclusions

In this article, we have described the optical phenomena altering *ic*OS data and outlined a workflow to correct them during data analysis. We have also presented a workflow to analyse *ic*OS data, and finally, a set of tools that can be used to achieve each step. A GUI is supplied with these tools to allow the analysis of *ic*OS data directly at the beamline or on the *ic*OS platform at ESRF so that the majority of the analysis can be done during a diffraction beam time. The *ic*OS app has already been successfully used in several TR-MX projects, including by non-experts or unsupervised users.

## Data Availability

The *ic*OS app, as well as instructions on how to use it, can be found at https://github.com/ncara/icOS.
